# Update in clinical management for gallbladder neuroendocrine carcinoma

**DOI:** 10.1097/MD.0000000000025449

**Published:** 2021-04-09

**Authors:** Hongwu Chu, Ying Shi, Junwei Liu, Dongsheng Huang, Jungang Zhang, Changwei Dou

**Affiliations:** a Hepatobiliary and Pancreatic Surgery, Zhejiang Provincial People's Hospital, Hangzhou Medical College, Hangzhou; b Qingdao University, Qingdao; c Obstetrics and Gynecology, Zhejiang Provincial People's Hospital, Hangzhou Medical College, Hangzhou, China.

**Keywords:** clinical feature, gallbladder, neuroendocrine carcinoma, treatment

## Abstract

**Background:**

Gallbladder neuroendocrine carcinoma (GB-NEC) is rare and there are few reports at present. We sought to review the current knowledge of GB-NEC and provide recommendations for clinical management.

**Methods:**

A systemic literature research was conducted in the websites of Pubmed, Medline, Web of Science, CNKI, Wanfang Data using the keywords including gallbladder combined with neuroendocrine carcinoma or neuroendocrine tumor or neuroendocrine neoplasm. Two reviewers independently screened the articles by reading the title, abstract and full-text.

**Results:**

In computed tomography (CT) and magnetic resonance imaging (MRI) examination, a well-defined margin, gallbladder replacing type with larger hepatic and lymphatic metastases could be helpful for differential diagnosis of GB-NEC and gallbladder adenocarcinoma (GB-ADC). Older age, unmarried status, large tumor size (>5 cm), positive margins, and distant Surveillance, Epidemiology and End result (SEER) stage are independently associated with poor survival. Surgical resection remains as the preferred and primary treatment. The potential survival benefit of lymphadenectomy for patients remains controversial. Platinum-based postoperative adjuvant chemotherapy may improve the survival. The efficacy of other treatments including immunotherapy, targeted therapy and somatostatin analogue needs further investigation.

**Conclusion:**

Typical imaging features could be helpful for preoperative diagnosis. Age, margin status, tumor size, marital status, histopathologic subtype and SEER stage may be independent predictors for the survival. Remarkable advances regarding the treatment for GB-NEC have been achieved in recent years. Further studies are needed to investigate the survival benefit of lymphadenectomy for patients with GB-NEC.

## 1 Introduction

Neuroendocrine neoplasms (NENs) are a group of rare and heterogeneous tumors originated from disseminated neuroendocrine cells with the incidence of about 6.98 per 100,000 people in 2012.^[1]^ They are more commonly found in the gastrointestinal tract and respiratory tract, accounting for 66% and 31% of all NENs, respectively.^[2]^ Primary gallbladder neuroendocrine neoplasms (GB-NENs) comprise only 0.5% of all NENs.^[3]^ According to 2019 World Health Organization (WHO) classification, NENs are classified into 3 main categories: well-differentiated neuroendocrine tumors (NETs), poorly-differentiated neuroendocrine carcinomas (NECs) and mixed endocrine non-endocrine neoplasms (MiNENs).^[4]^ Gallbladder neuroendocrine carcinoma (GB-NEC) encompasses small-cell and large-cell type with mitotic rate >20 mitoses/2 mm^2^ and/or Ki-67 index >20%.^[4]^ GB-NEC is extremely rare in clinical practice with few reports. Currently, the origin of GB-NEC remains unknown and is still under investigation. NENs can be divided into functional and non-functional neoplasms. Functional GB-NEC is able to secret peptides which can cause specific symptoms. However, unlike NENs from the gastrointestinal tract or respiratory tract, GB-NECs are mostly asymptomatic.^[5]^ Due to fact that GB-NEC is lack of special clinical manifestations and typical imaging features, it is difficult to distinguish GB-NEC from gallbladder adenocarcinoma (GB-ADC) without pathological examination. Additionally, there is currently no consensus on the surgical strategy and guidelines of GB-NEC. In clinical practice, the treatment of GB-NEC refers to GB-ADC. Radical surgical resection is considered the relatively optimal treatment.^[6]^ Compared with GB-ADC, GB-NEC is usually in advanced stage upon first diagnosis and associated with worse prognosis.^[7]^ However, high quality investigation regarding the pathogenesis, treatment modalities and prognosis of GB-NEC is currently unavailable. In this article, we retrospectively reviewed the clinical features, diagnosis, management and prognosis of GB-NEC.

## 2 Origin

There is no distribution of neuroendocrine cells in gallbladder tissues and the origin of GB-NEC is still under investigation. GB-NEC was proposed to be originated from the intestinal or gastric metaplasia of gallbladder epithelium. Chronic inflammation was believed to promote metaplastic changes of gallbladder epithelial cells to neuroendocrine cells, since NEC was mostly accompanied with cholelithiasis.^[3]^ It has been reported that intestinal metaplasia was presented in 11.7% of patients with cholelithiasis and 83.3% of them showed positive staining of chromogranin A (CgA), a specific marker of neuroendocrine cells.^[8]^ In the study performed by Chen et al,^[7]^ 8 of 10 patients with GB-NEC had the history of cholecystitis. On the other hand, some researchers insisted that GB-NEC was originated from the transformation of adenocarcinomas, since GB-NEC was often co-existed with adenocarcinomas.^[9–11]^ Additionally, it has been proved that NENs and adenocarcinoma could undergo inter-transformation in gastrointestinal tract.^[12]^ Furthermore, other researchers suggested that GB-NEC was derived from undifferentiated multipotent stem cells of gallbladder.^[3]^

## 3 Epidemiology

According to the Surveillance, Epidemiology and End result (SEER) database, the incidence of gallbladder neuroendocrine neoplasms (GB-NENs) was less than 0.74/100,000.^[13]^ As a type of poorly differentiated NENs, GB-NEC only accounts for 0.2% of all gastrointestinal NECs and 2.1% of all gallbladder carcinomas.^[13]^ In the study performed by Duffy et al,^[14]^ 13 of 435 patients with gallbladder carcinomas treated at the Memorial Sloan-Kettering Cancer Centre (MSKCC) were found to be NEC. According to the survey performed by Chen et al,^[7]^ GB-NEC accounted for 2.2% of gallbladder carcinomas. Recent studies have found that middle-aged and elderly female patients were at higher risk of developing GB-NEC. Chen et al^[7]^ reported the median age of GB-NEC was 61 years and the male to female ratio was 1:4. In another study of 8 GB-NEC patients, the median age was 60 years and the male to female ratio was 1:3.^[15]^ Cen et al^[1]^ collected the data of 248 GB-NEN cases from the SEER database, including 169 GB-NEC cases and the majority were female (70.4%), white (75.7%), and married (60.4%).

## 4 Pathological classification

Based on the WHO classification published in 2010, NENs are classified into 3 main categories: well-differentiated NETs, poorly-differentiated NECs and mixed adeno-neuroendocrine carcinomas (MANECs) (Table 1).^[16]^ MANECs are consisted of non-neuroendocrine carcinomas, usually ductal adenocarcinomas and acinar cell carcinomas, and NENs, and the proportion of each component is greater than 30%. Furthermore, according to the mitotic count and/or the Ki-67 index, NETs can be graded into grade 1, grade 2; NEC which includes large-cell type and small-cell type are graded into grade 3.^[16]^ However, in this classification, there is disconsistency between differentiation and grading. The tumor with high Ki-67 level which is graded into NECs may be well-differentiated.

**Table 1:** Pathological classification of neuroendocrine neoplasms: comparison between the editions of WHO 2010 and 2019.

WHO 2010	Ki-67 Index	Mitotic count /10 HPF	WHO 2019	Ki-67 Index	Mitotic rate (mitoses/2 mm^2^)
Well-differentiated NENs			Well-differentiated NENs		
NET grade 1	<3	<2	NET grade 1	<3	<2
NET grade 2	3–20	2–20	NET grade 2	3–20	2–20
			NET grade 3	>20	>20
Poorly differentiated NENs			Poorly differentiated NENs		
NEC grade 3	>20	>20	NEC grade 3	>20	>20
MANECs			MiNENs		

HPF = high power fields, MANEC = mixed adeno-neuroendocrine carcinoma, MiNEN = mixed endocrine non-endocrine neoplasm, NEC = neuroendocrine carcinoma, NEN = neuroendocrine neoplasm, NET = neuroendocrine tumor, WHO = World Health Organization.

Therefore, simply classifying according to the Ki-67 index and/or mitotic count will divide some well-differentiated tumors into NECs and can lead to inappropriate treatment.^[17]^ In 2019, WHO revised the classification system of tumors of digestive system.^[4]^ In this classification system, morphologically well-differentiated NETs with a high Ki-67 index (>20%) are defined as G3 NETs.^[18]^ It has been reported that the genomic features of G3 NETs resemble those of lower-grade NETs and differ from poorly-differentiated NECs.^[17]^ Well-differentiated NETs usually have mutations in MEN1, DAXX and ATRX, whereas NECs usually have TP53 or RB1 mutations.^[4]^ In addition, in the new classification system, MANECs are classified into MiNENs. This change is to reflect that not all MiNENs are high-grade malignancies and 1 or more of their components may belong to well-differentiated NENs category. Moreover, the non-neuroendocrine components of MiNENs are not necessarily all adenocarcinomas, but may also be squamous cell carcinomas.

## 5 Diagnosis

### 5.1 Clinical manifestation

According to whether the substance secreted by cancer cells causes typical clinical symptoms, GB-NEC can be divided into functional and non-functional. Functional GB-NEC is able to secret peptides such as serotonin and histamine which can cause specific symptoms, including diarrhea, flushing, edema and wheezing. Jin et al^[19]^ reported a 65-year-old patient with GB-NEC presented with flushing for 2 months and pathological specimens of the flushed skin showed that mucin was deposited between the collagen bundles in the dermis. However, the presence of carcinoid syndrome is very rare. We reviewed previously published studies and found that only a small proportion of reported cases had secretory symptoms due to the first-pass effect of liver. Patients were reported to suffer from distention, flushing and diarrhea while no edema was reported. Previous studies demonstrated that the main complaint was right epigastric discomfort, including pains with tenderness and distention.^[15]^ In addition, some patients also showed poor appetite, jaundice and weight loss. However, these non-specific symptoms of GB-NEC had no value for the differential diagnosis with GB-ADCs.^[7]^

### 5.2 Imaging and laboratory examination

With the development of imaging techniques, imaging examinations including contrast-enhanced computed tomography (CT), magnetic resonance imaging (MRI) and positron emission tomography/computed tomography (PET/CT) or PET/MRI revealed the typical imaging characteristics which is helpful to distinguish GB-NEC from other gallbladder diseases. Kim et al^[20]^ retrospectively analyzed the contrast-enhanced CT differentiation of GB-NENs from GB-ADCs and reported that a well-defined margin was more often observed in GB-NENs (94.7%, 18/19) than in GB-ADCs (10.6%, 2/19) (*P* < .0001). Bae et al^[21]^ also reported that, in MRI examination, well-defined margins, intact overlying mucosa were more frequently observed in GB-NENs than in GB-ADCs. Similar descriptions have been found in previous studies of gastrointestinal NENs.^[22,23]^ Researchers believed that NENs mostly were originated from the deep part of the lamina propria or submucosa, and thus the surface mucosal epithelium remained partially intact and was linearly enhanced. In addition, the sizes of metastatic lymph nodes (LNs) in GB-NENs were larger than those in adenocarcinomas.^[20,21,24]^ GB-NENs are more often manifested as gallbladder replacing type while adenocarcinomas as wall thickening type.^[20]^ It has been reported that PET/CT or PET/MRI was effective for the detection of NENs and helpful for identification of distant metastasis.^[25,26]^ Kamikihara et al^[27]^ reported a case preoperatively diagnosed with GB-NEC using somatostatin receptor scintigraphy (SRS). These preoperative imaging examinations are able to show the location of the lesions, suspected metastatic site and LNs status, and are valuable for determining the tumor-node-metastasis (TNM) staging and therapeutic strategy. Moreover, several typical imaging characteristics aided in differentiating between GB-NENs and other gallbladder disease.

Based on retrospective analysis of published literatures, no tumor marker was found to be specific for the diagnosis of GB-NEC. In the study performed by Kamboj et al,^[28]^ the positive rate for cancer antigen 199 was 57.1% (8/14), followed by 25.0% for carcinoembryonic antigen (CEA, 2/8). Study performed by Chen et al^[7]^ showed that the positive rates for CEA, CA-199 and cancer antigen 125 were 57.1% (4/7), 25.0% (2/8), and 12.5% (1/8), respectively.

### 5.3 Immunohistochemistry

The confirmative diagnosis of GB-NEC requires pathology and immunohistochemistry, including CgA, synaptophysin (Syn), neuro-specific enolase (NSE), epithelial membrane (EMA), and cytokine (CK). CgA and Syn are regarded as specific biomarkers. In addition, the plasma of CgA is thought to be related to tumor burden, there for it may be used to monitor efficacy and detect recurrence.^[29]^ Chen et al^[7]^ reported the positive rates of CgA and Syn were 100% (10/10) and 88.9% (8/9), respectively. In another study, CgA and NSE had positive rates of 91.9% and 84.8%, respectively.^[30]^ Monier et al^[31]^ suggested that urinary 5-hydroxyindole acetic acid (5-HIAA) was valuable for the diagnosis of GB-NEC. However, the positive rate of 5-HIAA was not high since most GB-NECs were non-functional and secretion of 5-HIAA secretion was insufficient or absent in most patients.

## 6 Treatment

### 6.1 Surgery

Due to the malignant potential of GB-NEC, surgical resection remains as the preferred and primary treatment, including simple cholecystectomy, palliative cholecystectomy, radical cholecystectomy.^[32]^ The patients undergoing surgery had better survival than patients without surgery.^[1]^ It has been reported that simple cholecystectomy could be performed for in situ GB-NENs and those of T1N0M0 stage.^[3]^ In the study performed by Liu et al,^[33]^ all 3 patients with GB-NEC were in the stage of T1bN0M0 and underwent laparoscopic cholecystectomy with gallbladder bed cautery without any postoperative chemotherapy or radiotherapy. No recurrences were found during the follow-up period of 26 months. For patients with GB-NEC in more advanced stage, radical resection (including cholecystectomy, resection of local liver and regional LNs dissection) is beneficial to those without distant metastasis. Compared with other treatment modalities, radical resection could promote longer survival time in patients with GB-NEC.^[6]^ With the improvement of surgical techniques, laparoscopic radical resection for GB-NEC is increasing. Kim et al^[34]^ reported a patient with GB-NEC diagnosed at T3N1M0 stage underwent laparoscopic radical cholecystectomy and combined chemoradiation therapy, and no evidence of recurrence during the follow-up period of 14 months. Elahi et al^[35]^ reported a 52-year-old woman with GB-NEC with involvement of the LNs and omentum underwent laparoscopic radical cholecystectomy with no complications were observed during the recovery, and received chemoradiation therapy after surgery, with a survival time of more than 46 months. For patients with distant metastasis, the value of surgery remains controversial. Palliative cholecystectomy can reduce tumor burden, facilitate the implementation of postoperative treatments and might improve the quality of life. GB-NEC had high lymphatic metastases.^[28,36]^ Moreover, Chen study reported that GB-NEC had higher N2 lymphatic metastases rate than adenocarcinoma (70.0 vs 34.0%; *P* < .05), and lymphatic metastases can occur at early stage.^[7]^ However, due to the lack of large, prospective, randomized clinical trial data, the potential survival benefit of lymphadenectomy for patients with GB-NEC remains controversial. Cen et al^[1]^ reported that to the patients with GB-NEN who underwent gallbladder surgery, the further addition of lymphadenectomy has no effect on survival. However, that study included some patients with highly differentiated GB-NET (29.4%), and the lymphatic metastases rate was only 29.4%.^[1]^ For patients with gallbladder carcinomas, resection of 6 or more LNs was associated with improved survival, but patients with confirmed N2 lymphatic metastases do not benefit from radical resection.^[37,38]^ Considering the high N2 lymphatic metastases rate of GB-NEC, N2 level nodes biopsy may help improve prognosis and may be used to develop surgical approaches.^[39]^ In addition, further studies are needed to confirm survival benefit of lymphadenectomy with different number of regional LNs for patients with GB-NEC.

### 6.2 Chemotherapy

Since GB-NEC is highly malignant and progresses rapidly, many patients are in a late stage and lost the opportunity of radical surgery. Chemotherapy is critical for these patients. The platinum-based chemotherapy regimens were used for the treatment of GB-NECs and achieved satisfactory responses.^[40]^ In the study performed by Kanetkar et al,^[41]^ 6 patients of locally advanced GB-NEC received 3 cycles of new adjuvant chemotherapy (NACT) with the program of carboplatin plus etoposide and the tumor volume was subsequently reduced. More importantly, 5 out of 6 patients underwent radical cholecystectomy after NACT and showed negative margins (R0).^[41]^ Chen et al^[7]^ reported that compared with patients who only received surgical resection, patients who received postoperative chemotherapy and radiotherapy had better prognosis, with the median survival time of 3.0 months versus 12.7 months. Programmed death 1 inhibitor nivolumab in refractory small-cell lung cancer and the programmed death ligand 1 inhibitor avelumab in metastatic Merkel-cell carcinoma are checkpoint inhibitors approved by the Food and Drug Administration for high-grads NECs. However, there are few reports on the use of checkpoint inhibitors in GB-NECs. Chorath et al^[42]^ presented a case of 52-year-old woman with metastatic GB-NEC experiencing durable response to carboplatin, etoposide, nivolumab, and ipilimumab, that highlights that adding checkpoint blockade to standard platinum-based chemotherapy may be an effective option for metastatic GB-NECs.

### 6.3 Targeted therapy

Two targeted drugs including the tyrosine kinase inhibitor sunitinib and the rapamycin inhibitor everolimus, have been demonstrated to prolong the progression free survival for patients with advanced pancreatic neuroendocrine neoplasms (pNENs).^[43]^ Vascular endothelial growth factor (VEGF) and its receptor were highly expressed in NENs and VEGF-mediated angiogenesis played an important role in the pathogenesis, progression, metastasis and recurrence of NETs.^[44]^ Wang et al^[45]^ reported that sunitinib was beneficial for the patients with pNENs, with the objective response rate of 5.0%, and the stable disease rate of 81.7%. In the study performed by Lee et al,^[46]^ 16 patients with advanced pNENs were treated with sunitinib which showed significant clinical benefit with the objective response rate and clinical benefit rate of 44% and 69%. Additionally, PI3K/AKT/mTOR signaling pathway was found to play an important role in the pathogenesis and progression of NENs.^[47–50]^ Pusceddu et al^[51]^ reported that everolimus, a direct inhibitor of PI3K/AKT/mTOR signaling pathway, showed remarkable efficacy for well differentiated NENs. This drug showed acceptable tolerability, with most adverse events being of mild or moderate severity. However, more investigations are necessary to further confirm the value of sunitinib and everolimus for the treatment of GB-NECs.

### 6.4 Somatostatin analogue

Endocrine therapy for NENs is currently under intensive investigation due to the endocrine-dependent biological behavior of these tumor cells. Somatostatin analogues including octreotide, lanreotide, and long-acting agent Sandostatin LAR have suppressive effect on the proliferation of tumor cells. Some studies have shown that octreotide inhibited tumor progression and improved patients’ symptoms and prognosis.^[52,53]^ For patients with positive expression of somatostatin receptor, somatostatin analogues, especially the long-acting agent, can be used as a new adjunct therapy.

## 7 Prognosis

GB-NEC is highly malignant and aggressive, and progresses rapidly with poor prognosis. Systemic metastasis is not a rare event, even for those in early stage. Most patients have metastasis at the time of treatment, and LNs are the commonest metastatic site, followed by liver.^[28,36,54]^ Study by Kamboj et al^[28]^ showed a median survival of 3 months (ranging from 1 to 9.5 months), while Lee et al^[55]^ reported a median survival of 8 months. Compared with GB-ADC, the prognosis of GB-NEC is relatively worse. The median survival time of 13 patients with GB-NEC was 9.8 months (95% CI 5.3–20.1 months), while the median survival time of the patients with gallbladder carcinomas was 10.3 months (95% CI 8.8–11.8 months).^[14]^ Chen et al^[7]^ reported a median survival time of 3 months with 1-, 2-, and 3-year survival rates of 20, 10, and 0%, while the median survival time of the 377 GB-ADC patients treated with the same period was 6 months with 1-, 2-, 3-, and 5-year survival rates of 38.0%, 31.0%, 30.1%, and 28.4%. However, Yun study did not support this hypothesis, with the overall five-year survival rate of GB-NEC higher than GB-ADC.^[56]^ Suspecting that confounding factors between the 2 groups might affect the result, Yan et al^[5]^ used propensity score matching to reduce the influence of confounding factors between the 2 groups, and the results still showed that GB-NEC had worse prognosis than GB-ADC. The comparison of these studies was shown in Table 2. Due to the scarcity of these tumors, the risk factors of GB-NEC patients remain unclear. Cen et al^[1]^ performed a multivariate survival analysis on 248 GB-NEN patients, including 169 GB-NEC patients, and the results showed that older age, large tumor size (>5 cm), unmarried status, and distant SEER stage were important independent predictors of decreased overall survival time and cancer-specific survival time (*P* < .05). Ayabe et al^[57]^ also reported that older age, positive margins, and large-cell type are independently associated with poor survival after resection.

**Table 2:** Comparison of published studies of gallbladder neuroendocrine carcinoma.

First author [Ref.]	Chen Chen^[7]^	Anirban Maitra^[36]^	Jae-Myeong Lee^[55]^	Meenakshi Kamboj^[28]^	Shida Yan^[5]^
No. of cases	10	12	12	19	15
TNM stage					
I-II	1	NA	NA	0	8
III	0	NA	NA	0	3
IV	9	NA	NA	19	4
Histopathologic subtype					
NETs	NA	0	1	0	0
NECs	NA	6	6	18	10
MANECs	NA	6	5	1	5
Metastasis	LNs 7	LNs 5, Liver 4, Periportal soft tissue 3	NA	LNs 13, Liver 12, Omentum/Peritoneum 4, Ascites 3, Bone 2	NA
Median survival time (mo)	3.0 (1.8–23.3)	9.0 (3.0–25.0)	8.0	3.0 (1.0–9.5)	20.4 (0.5–40.0)

LN = lymph node, MANEC = mixed adeno-neuroendocrine carcinoma, NA = not available, NEC = neuroendocrine carcinoma, NET = neuroendocrine tumor, TNM = tumor-node-metastasis.

Our research also has limitations. Firstly, there are few studies on GB-NEC, and the sample size of these studies is small. The basic characteristics (such as race, sex, age, disease stages, treatment methods etc.) of patients in the different studies included in this article are different. Secondly, we only conducted retrospective descriptive studies, and did not conduct statistical analysis. Therefore, prospective studies with larger samples are needed to prove the generality of our conclusions.

## 8 Conclusion

**Figure 1:**
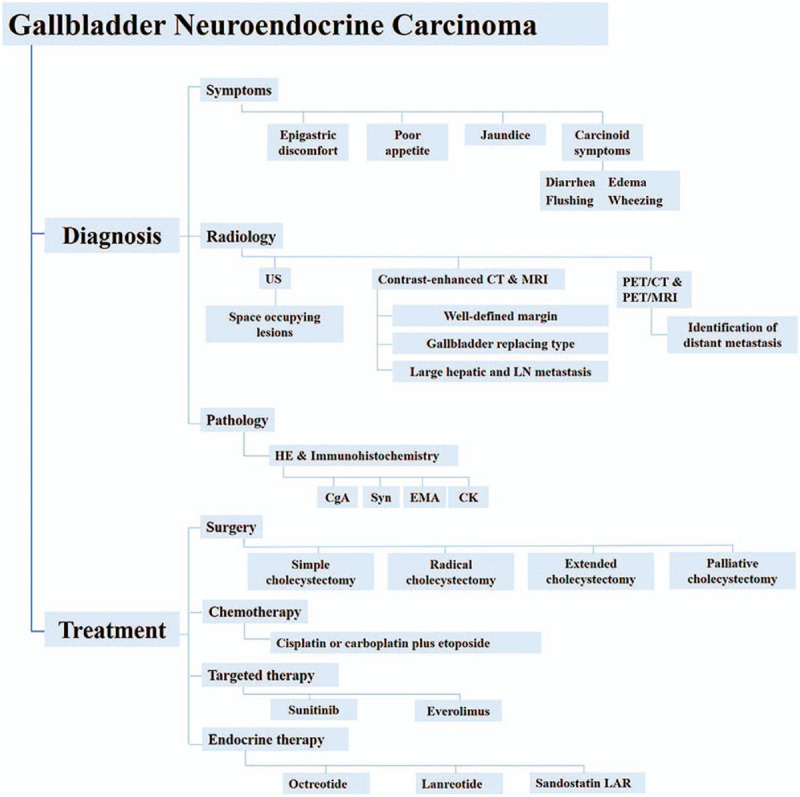
Diagnosis and treatment diagram for gallbladder neuroendocrine carcinoma. CgA = chromogranin A, CK = and cytokine, CT = computed tomography, EMA = epithelial membrane, HE = hematoxylin-eosin, LN = lymph node, MRI = magnetic resonance imaging, PET = positron emission tomography, Syn = synaptophysin, US = ultrasound.

In this study, we reviewed the clinical management of GB-NEC (summarized in Fig. 1) and demonstrated the updated knowledge of GB-NEC based on recently published literature. GB-NEC, a rare and highly malignant disease in clinical practice, has relatively higher incidence in middle-aged and elderly female patients. The combined use of contrast-enhanced CT, MRI, SRS, PET/CT or PET/MRI and the typical imaging revealed by these imaging examinations could be helpful for preoperative diagnosis. Confirmative diagnosis of GB-NEC still requires pathology and immunohistochemistry examinations. GB-NEC has poor prognosis, even worse than that of GB-ADCs. Age, margin status, tumor size, marital status, histopathologic subtype and SEER stage may be independent predictors for the survival of patients with GB-NEC. Surgical resection remains the first-line therapeutic option for GB-NEC and can improve survival. More studies are needed to further investigate the survival benefit of LNs dissection for patients with GB-NEC. Platinum-based postoperative adjuvant chemotherapy may be helpful to improve the prognosis for those in advanced stages. The efficacy of other treatments including immunotherapy, targeted therapy and somatostatin analogue needs further investigation. In clinical practice, both patient's condition and the efficacy of different therapeutic options should be taken into consideration for prescribing optimal individualized management.

## Author contributions

**Conceptualization:** Changwei Dou.

**Data curation:** Hongwu Chu.

**Formal analysis:** Junwei Liu.

**Funding acquisition:** Jungang Zhang.

**Investigation:** Hongwu Chu, Ying Shi.

**Methodology:** Ying Shi.

**Software:** Hongwu Chu, Junwei Liu.

**Writing – original draft:** Hongwu Chu.

**Writing – review & editing:** Dongsheng Huang, jungang zhang, Changwei Dou.
